# Pegylated liposomal doxorubicin (PLD) in daily practice—A single center experience of treatment with PLD in patients with comorbidities and older patients with metastatic breast cancer

**DOI:** 10.1002/cam4.6041

**Published:** 2023-05-06

**Authors:** T. Wallrabenstein, E. Daetwyler, A. Oseledchyk, C. Rochlitz, M. Vetter

**Affiliations:** ^1^ Medical Oncology University Hospital Basel Basel Switzerland; ^2^ Hematology/Oncology University Medical Center Freiburg Freiburg Germany; ^3^ Medical Oncology Kantonsspital Baselland Liestal Switzerland

**Keywords:** comorbidity, elderly, metastatic breast cancer, pegylated liposomal doxorubicin, real‐world data

## Abstract

**Purpose:**

Real‐world data about pegylated liposomal doxorubicin (PLD) in patients with metastatic breast cancer (MBC) are limited. We have aimed to highlight the role of PLD in daily practice focusing on older patients and patients with comorbidities with MBC.

**Methods:**

We analyzed electronic records of all patients with advanced/metastatic breast cancer treated with single‐agent PLD at the University Hospital Basel between 2003 and 2021. Primary endpoint was time to next chemotherapy or death (TTNC). Secondary endpoints were overall survival (OS), progression‐free survival (PFS), and overall response rate (ORR). We performed univariate and multivariate analysis for clinical variables.

**Results:**

112 patients with MBC having received single‐agent PLD in any treatment line were analyzed, including 34 patient who were older than 70 years and 61 patients with relevant comorbidities. Median TTNC, OS, and PFS for treatment with PLD were 4.6, 11.9, and 4.4 months, respectively. ORR was 13.6%. Age >70 years predicted shorter OS (median 11.2 months) in multivariate analysis (hazard ratio [HR] 1.83, 95% CI 1.07–3.11, *p* = 0.026). Age and comorbidities did not significantly affect other endpoints. Unexpectedly, hypertension predicted longer TTNC (8.3 months, *p* = 0.04) in univariate analysis, maintained in multivariate analysis as a trend for both TTNC (HR 0.62, *p* = 0.07) and OS (HR 0.63, *p* = 0.1).

**Conclusion:**

Age predicted shorter OS significantly but median OS was not relevantly shorter in older patients. PLD remains a treatment option in patients with comorbidities and older patients with MBC. However, our real‐world results of PLD appear underwhelming compared to relevant phase II trials through all age groups, pointing to an efficacy‐effectiveness gap, possibly due to sampling bias.

## Graphical abstract

We have examined the efficacy of single‐agent pegylated liposomal doxorubicin (PLD) in a real‐world population of patients with metastatic breast cancer (MBC) and in the subgroups of elderly patients and patients with comorbidities. We found no significant inferiority of PLD in these more vulnerable patient groups than in the overall population; however, substantially lower response rates in the overall population as compared to previous prospective evidence.

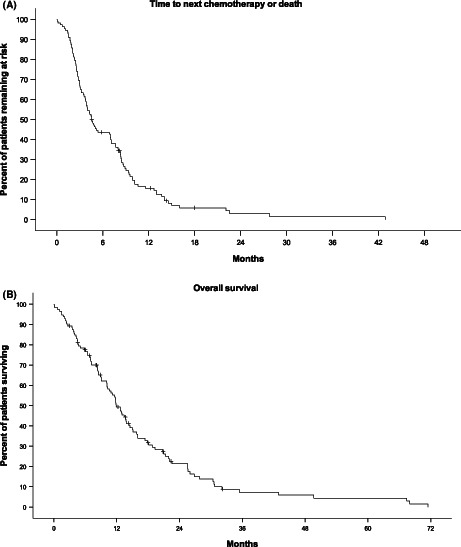

## 1 INTRODUCTION

Breast cancer (BC) is a major global health care burden. In Switzerland alone, more than 6200 women are diagnosed with breast cancer every year, accounting for one third of all new cancer cases among women. Breast cancer is the most frequent cancer‐related cause of death in women in Switzerland.^1^ Nowadays, around 20%–30% of patients will have metastatic relapse.^2^

Treatment of metastatic BC remains a challenge to this day. Treatment choices depend on disease biology, pretreatment, age, comorbidities, patient preferences, and many other factors. Chemotherapy has long been the backbone of treatment in endocrine resistant BC. More recently antibody‐drug‐conjugates (ADCs) have become available as 2nd line options in Her‐2‐positive and triple negative BC.^3^, ^4^ Sequential single‐agent chemotherapies were found to have comparable efficacy and lower toxicity than multiple‐agent regimens and have become standard‐of‐care except in patients with high tumor burden or visceral disease.^5^ According to current guidelines, options for first line therapy in Her2 negative metastatic breast cancer (MBC) include anthracyclines (doxorubicin, liposomal doxorubicin), taxanes (paclitaxel, docetaxel), anti‐metabolites (capecitabine, gemcitabine), and microtubule inhibitors (vinorelbine, eribulin). Further options are available for patients with germ‐line BRCA‐mutations or PD‐L1‐positive triple negative breast cancer (TNBC).^6^, ^7^, ^8^ Second‐line options include all of the above. Additional second‐line options for patients with TNBC include platinum derivates. Her2 positive MBC should be treated with a Her2‐directed first line therapy, usually combined with chemotherapy.^9^ In patients with ER+/HER2‐negative disease first‐line standard‐of‐care includes an aromatase inhibitor or SERD plus a CDK4/6 inhibitor.

Treatment guidelines rest upon evidence gathered in clinical phase II/III trials. However, the realities of daily clinical practice often differ from trial settings. Trial inclusion criteria are restrictive so that many patients seen in daily practice do not fit these criteria. Nevertheless, there is a need to offer treatment to these patients and treating physicians make individual decisions together with their patients. Pegylated liposomal doxorubicin (PLD) is an alternative to conventional anthracyclines in patients with increased cardiac risk, previous exposure to anthracyclines, and in older patients with breast cancer. PLD was found to have similar efficacy, yet lower rate of cardiac events, alopecia, nausea, and myelosupression than single‐agent conventional doxorubicin, however a higher rate of palmar–plantar erythrodysesthesia (PPE).^10^ Phase II trials and prospective observational studies have reported response rates as high as 30%–43%; however, lower numbers were reported in older patients and pretreated patients (Table 1).^11^, ^12^, ^13^, ^14^, ^15^, ^16^, ^17^

**TABLE 1:** Overview of relevant phase II trials investigating single‐agent pegylated liposomal doxorubicin in patients with metastatic breast cancer.

	Al‐Batran (2006)^13^	Al‐Batran (2006)^14^	Coleman (2006)^11^	Ranson (1997)^12^	Falandry (2013)^15^
Number of patients	46	79	116	71	60
Treatment line	2nd or 3rd	2nd or later	1st or 2nd	—	1st
Inclusion criteria					
Performance status	≥70%	≥70%	ECOG≤2	≥60%	—
Age (years)	>18	>18	>65	>60	>70
Receptor status	All	All	All	All	Her2‐
Patient criteria					
Median age (years)	60	58	69	57	77
≥3 sites of metastasis (%)	35	35	58	39	—
Her2‐positive (%)	11	4	—	—	0
HR‐positive (%)	74	86	59	—	87
Visceral disease (%)	—	83	—	70	73
Endocrine therapy (%)	85	100	79	80	72
Adjuvant (%)	—	—	13	—	—
Previously in MBC (%)	—	100	33	—	—
Prior chemotherapy (%)	—	100	33	—	22
Adjuvant (%)	—	—	22	—	22
In MBC (%)	65	100	14	39	—
Dosing (mg/m^2^)	40, q4w	50, q4w	50/60, q4/6w	45–60, q3‐4w	40, q4w
Outcome measures					
ORR	13%	12.7%	29%–31%	31%	20%
CBR	48%	40.5%	63%–64%	62%	80%
PFS (months)	3.3	3.6	5.4–5.8	—	6.1
OS (months)	10.7	12.3	—	7	15.7

Abbreviations: AT, anthracycline treatment; CBR, clinical benefit rate; ECOG, eastern cooperative oncology group performance status; Her2, human epidermal growth factor receptor 2; Her2‐, Her2 negative; HR, hormone receptor; HR‐, HR negative or endocrine resistant; m^2^, square meter; mg, milligram; ORR, objective response rate; OS, overall survival; PFS, progression‐free survival; q4w, every 4 weeks.

While previous evidence for PLD has been contradictory likely due to heterogeneous populations, our general observation was that the reported results of clinical and observational trials are hardly reproducible in daily clinical practice. We have thus presumed an efficacy‐effectiveness gap due to a sampling bias with a disadvantage toward the actual patient clientele seen in daily practice, which we assumed to be older and more morbid than trial populations. Our question was whether vulnerable patients really benefit from PLD. In order to investigate this question, we have aimed to provide a comprehensive treatment experience by analyzing *all* patients with MBC treated with PLD at our center. We have aimed to characterize our patient clientele, assess outcome measures, and to perform subgroup analyses in patients we presumed to be at disadvantage, namely older patients, patients with comorbidities and patients who have received previous adjuvant and/or palliative chemotherapy.

## 2 PATIENTS AND METHODS

### 2.1 Study design and patient population

We retrospectively identified by keyword search *all* patients with MBC having received single‐agent PLD at the University Hospital Basel, Switzerland between July 01, 2003 and May 31, 2021. Electronic patient charts were viewed, and clinical data points were collected in an anonymized database. Last update was on January 31, 2022.

Inclusion criteria for this retrospective study were a diagnosis of MBC with histopathological confirmation and radiologically confirmed advanced/metastatic disease. Patients must have received at least one dose of PLD for MBC at our hospital.

This study was approved by the responsible ethics committee, Ethikkommission Nordwest‐und Zentralschweiz (EKNZ, Switzerland) on July 21, 2021 (project ID 2021–00709). Patient consent regarding use of health‐related data for research purposes was not available from all patients due to the retrospective nature of our investigation. The requirement for informed consent has been waived by the ethics committee in these cases.

### 2.2 Endpoints

The primary endpoint was time to next chemotherapy or death (TTNC), defined as time from treatment initiation until initiation of subsequent line of chemotherapy or death from any cause. We have selected TTNC as clinically meaningful primary endpoint for our retrospective analysis. Even though this endpoint is not commonly reported in clinical trials—thus impeding comparability—we consider it superior to PFS because treatment failures for other reasons than progression (e.g., clinical deterioration, toxicity, or patient preference) are implicated.

Secondary endpoints were overall survival (OS), progression‐free survival (PFS), and objective response rate (ORR). OS was defined as time from treatment initiation until death from any cause. PFS was defined as time from treatment initiation until radiographic progression or death from any cause. Patients who were lost to follow‐up were censored at the time of last contact regarding all time‐to‐event endpoints. Response data were taken from routine CT‐scans and was defined according to RECIST 1.1 criteria.^18^ If RECIST criteria were unavailable for response evaluation in single patients due to the retrospective nature of our study, no progression was documented, unless unequivocal clinical progression has been recorded by the treating physician (e.g., new cutaneous metastasis).

### 2.3 Statistical analysis

Time‐to‐event endpoints were measured from beginning of therapy and calculated by Kaplan–Meier method. Confidence intervals for time‐to‐event endpoints were based on z‐values. Confidence intervals for categorical endpoints (ORR) were calculated by Clopper‐Pearson exact method. We performed univariate (logrank) and multivariate analysis to determine predictive factors. Multivariate analyses were calculated by Cox regression on time‐to‐event endpoints and by binary logistic regression on discrete endpoints. Predefined variables were age (<70 vs. ≥70 years), relevant comorbidity (defined as congestive heart failure, coronary heart disease, hypertension, chronic obstructive/restrictive pulmonary disease, chronic kidney failure, diabetes, or other malignant disease), ER‐status, PR‐status, Her2‐status, tumor grade, syn−/metachronous metastatic disease, and line of treatment (first line vs. later line). Subgroups with less than a minimum of 10 patients were excluded from analysis. Significance level was selected at 0.05 and no correction was applied for multiple statistical testing. All statistical analyses were performed with SPSS version 28 (IBM Corp.).

## 3 RESULTS

### 3.1 Patients and disease characteristics

112 patients with MBC treated with single‐agent PLD at the University Hospital Basel between July 2003 and May 2021 were retrospectively identified. Median age was 62 years at the time of treatment initiation with PLD (Table 2). 34 patients (30%) were 70 years or older and 61 patients (54%) had significant comorbidities with an overlap of 27 patients (24%). Most predominant comorbidities were arterial hypertension (28%), heart disease (9%), and diabetes (7%). Most patients (82%) had hormone receptor positive BC. 36 patients (32%) had metastatic disease when first diagnosed with breast cancer, the other 76 patients (68%) had locoregional disease when first diagnosed with BC (early BC) have received curative treatment and have developed metastatic disease at a later time. 55% of patients had visceral disease when first diagnosed with MBC. 45 of all patients (40%) have received previous neoadjuvant or adjuvant chemotherapy, including 34 patients (30%) who have received conventional anthracylines.

**TABLE 2:** Baseline‐, disease‐, and treatment characteristics of 112 patients with metastatic breast cancer treated with single‐agent pegylated liposomal doxorubicin (PLD).

Characteristic	Patients (*n* = 112)	Patients ≥70 years (*n* = 34)	Patients with comorbidities (*n* = 61)
Median age at treatment initiation	62.3	76.2	67
Relevant comorbidity	61 (54)	27 (79)	
Arterial hypertension (%)	31 (28)	16 (47)	31 (51)
Heart/Cardiovascular (%)	10 (9)	7 (21)	10 (16)
Diabetes (%)	8 (7)	3 (9)	8 (13)
Pulmonary (%)	6 (5)	3 (9)	6 (10)
Other (%)	33 (29)	13 (38)	33 (54)
Receptor status			
ER positive (%)	90 (80)	31 (91)	51 (84)
PR positive (%)	76 (68)	26 (76)	46 (75)
Her‐2 positive (%)	11 (10)	1 (3)	5 (8)
Triple negative breast cancer (%)	14 (13)	3 (9)	7 (11)
M‐stage at primary diagnosis			
M0/cMx (%)	76 (68)	22 (65)	39 (64)
M1 (%)	36 (32)	12 (35)	22 (36)
Site at diagnosis of MBC			
Bone (%)	77 (69)	23 (68)	42 (69)
Lung (%)	37 (33)	13 (38)	25 (41)
Liver (%)	36 (32)	6 (18)	18 (30)
Pleural (%)	12 (11)	6 (18)	10 (16)
CNS (%)	9 (8)	1 (3)	4 (7)
Peritoneal/abdominal (%)	7 (6)	4 (12)	3 (5)
Other sites (%)	52 (46)	16 (47)	28 (46)
Visceral disease (%)	62 (55)	19 (56)	36 (59)
1–2 organs involved (%)	74 (66)	24 (71)	41 (67)
≥3 organs involved (%)	38 (34)	10 (29)	20 (33)
Primary treatment			
Breast conservative surgery (%)	53 (47)	16 (47)	27 (44)
Breast ablative surgery (%)	34 (30)	13 (38)	19 (31)
Sentinel node resection (%)	34 (30)	9 (26)	14 (23)
Axillar revision (%)	62 (55)	22 (65)	33 (54)
Neoadjuvant chemotherapy			
Any (%)	10 (9)	1 (3)	1 (2)
Neoadjuvant taxanes (%)	9 (8)	1 (3)	1 (2)
Neoadjuvant anthracycline (%)	9 (8)	0 (0)	0 (0)
Adjuvant chemotherapy			
Any (%)	35 (31)	5 (15)	18 (30)
Taxane (%)	19 (17)	3 (9)	8 (13)
Anthracycline (%)	25 (22)	3 (9)	12 (20)
Adjuvant endocrine therapy			
Any (%)	54 (48)	18 (53)	29 (48)
Minimum of 5 years (%)	25 (22)	9 (26)	17 (28)
Radiotherapy			
Adjuvant RT (%)	66 (59)	18 (53)	32 (52)
RT for MBC before first line (%)	28 (25)	15 (44)	33 (54)
Systemic treatment for MBC			
Endocrine therapy before first CT (%)	69 (62)	25 (74)	43 (70)
Median total lines (average, range)	5 (4.8, 1–1)	4 (5, 1–11)	4 (5, 1–11)
Median lines after PLD (average, range)	1 (1.4, 0–3)	0 (1, 0–3)	1 (1, 0–5)
Bisphosphonate/denosumab (%)	64 (57)	25 (74)	41 (67)

Abbreviations: CNS, Central Nervous System; CT, chemotherapy; ER, estrogen receptor; Her‐2, human epidermal growth factor receptor 2; MBC, metastatic breast cancer; PR, progesterone receptor; *n*, number; RT, radiotherapy.

### 3.2 Treatment characteristics

Patients have received a median number of five treatment lines for MBC, including endocrine and antineoplastic lines of treatment. PLD was first line chemotherapy in 52 patients (46%), second line in 31 patients (28%), and third or later line in 29 patients (26%). 69 patients (62%) have received previous endocrine treatment for MBC before starting PLD, including 29 patients who have received previous cdk4/6‐inhibitors. 65 patients (58%) had at least one subsequent line of chemotherapy after PLD. 43 patients (38%) had no further treatment after PLD, and 4 patients (4%) had only endocrine treatment after PLD.

### 3.3 Outcome parameters

**FIGURE 1:**
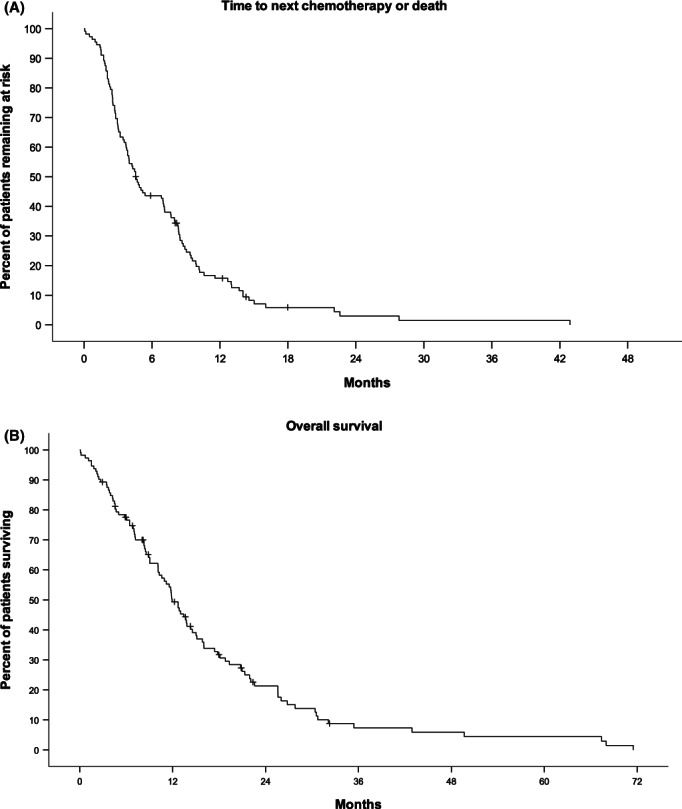
Time to next chemotherapy (A) and overall survival (B) of 112 patients with metastatic breast cancer treated with single‐agent pegylated liposomal doxorubicin (PLD) at the University Hospital Basel. Median time to next chemotherapy (A) was 4.6 months (95% confidence interval 3.6–5.6 months) and median overall survival (B) was 11.9 months (95% confidence interval 1.0–13.9 months).

Median TTNC was 4.6 (95% CI 3.6–5.6, Figure 1A), median OS 11.9 (95% CI 1.0–13.9, Figure 1B), and median PFS 4.4 (95% CI 3.3–5.5) months in our total population, respectively. Of 112 patients, 103 were evaluable for response. 14 patients had a partial remission, 36 had stable disease, 42 had progressive disease, and 11 patients had early death. ORR was 13.6% (95% CI 7.6–21.8) and clinical benefit rate was 48.5% (95% CI 38.6–58.6). There was no difference between patients having received PLD in the first line or later regarding TTNC (4.2 vs. 4.6 months, *p* = 0.83), OS (13.7 vs. 11.7 months, *p* = 0.72), PFS (4.0 vs. 4.8 months, *p* = 0.87), and ORR (16% vs. 10%, *p* = 0.41).

### 3.4 Subgroup analysis

**FIGURE 2:**
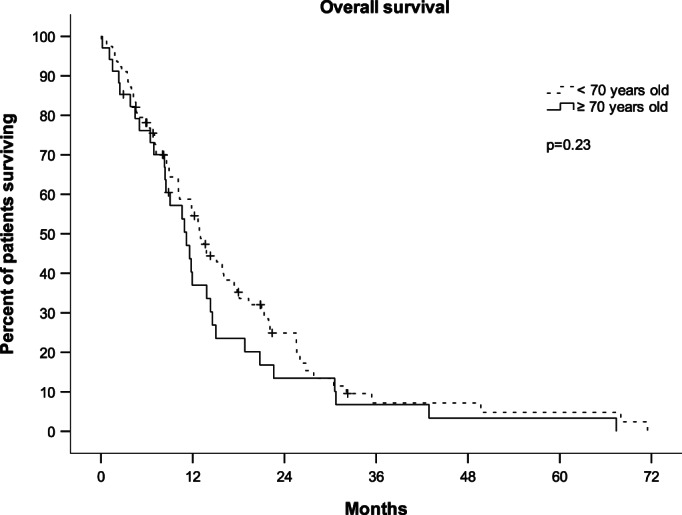
Overall survival of 112 patients with metastatic breast cancer treated with single‐agent pegylated liposomal doxorubicin (PLD) at the University Hospital Basel according to age at the time of treatment initiation (below or above 70 years). Median overall survival was 11.2 months (95% confidence interval 7.9–14.5 months) in patients who were 70 years and older compared to 11.9 months (95% confidence interval 1.0–13.9 months) in patients who were younger than 70 years. *p = p‐value*, calculated by logrank test.

Outcome measures for patients who were 70 years or older did not significantly differ from patients <70 years in univariate analysis (Figure 2). However, age was significantly associated with shorter OS in multivariate analysis (HR 1.83, 95% CI 1.07–3.11, *p* = 0.026). Age did not significantly affect TTNC, PFS, or ORR in univariate or multivariate analysis. Median TTNC in patients who were 70 years or older was 5.0 months (95% CI 0–10.4) as compared to 4.6 (95% CI 3.6–5.6) in the overall population and median OS was 11.2 months (95% CI 7.9–14.5) compared to 11.9 months (95% CI 1.0–13.9).

Comorbidities were not significantly associated with an adverse outcome. Unexpectedly, hypertension was associated with longer TTNC in univariate analysis (8.3 months, 95% CI 6.3–10.3, as compared to 4.6 months in the overall population, *p* = 0.04). This effect was maintained in multivariate analysis as a trend for both TTNC (HR 0.62, 95% CI 0.37–1.05, *p* = 0.07) and OS (HR 0.63, 95% CI 0.36–1.10, *p* = 0.1).

Patients with progesterone receptor‐positive BC had significantly, yet irrelevantly longer OS (12.9 vs. 11.9 months, *p* = 0.05), an effect that was maintained in multivariate analysis only as a trend (hazard ratio [HR] 0.57, 95% CI 0.29–1.12, *p* = 0.1). Other disease characteristics did not significantly affect any endpoint. Results of further univariate and multivariate subgroup analyses are reported in Tables S1–S3.

## 4 DISCUSSION

Real‐world evidence for treatment with PLD in patients with MBC is limited and here we report a first retrospective and comprehensive single‐center treatment experience. Observed outcome measures for single‐agent PLD (median TTNC 4.6 months, median PFS 4.4 months, median OS 11.9 months and ORR 14%) were underwhelming, and comparing to published phase II and prospective observational trials, our real‐world treatment results were inferior (Table 1).^16^, ^17^ We hypothesized this to be due to a higher median age of our population (62 years). However, two phase II trials with a specifically geriatric population and a median age of 69–77 years have reported ORR in the range of 20%–30%.^11^, ^15^ Unlike our population, all patients in these trials have received PLD in the first line. Next, we hypothesized whether our real‐world results might be inferior to previously published data because more than half of all patients included in our study population have received PLD as second or later line chemotherapy. Indeed, there are two phase II trials by Al‐Batran that have investigated PLD as second‐ or third‐line chemotherapy and have reported results that are more corresponding to ours.^13^, ^14^ However, we found no significant difference in our study group between patients who have received PLD in first‐line chemotherapy or later.

In summary, we interpreted our inferior results in terms of an efficacy‐effectiveness gap between the conditions of clinical trials and daily practice as well as due to our heterogeneous real‐world population that has included a large fraction of older patients, patients with comorbidities, and patients with previous treatment lines. It must be assumed that real‐world patient populations have a higher morbidity than the selected populations of clinical trials. Also, compliance is likely higher under trial conditions. In addition to such a sampling bias, there can also be an additional treatment bias. It is likely that mono‐PLD is frequently the treatment of choice in less fit patients.

In our analysis, age predicted shorter OS in multivariate analysis with statistical significance. However, this difference was small and therefore not clinically meaningful (median OS of 11.2 months compared to 11.9 months, Figure 2). Neither age nor comorbidities were adversely associated with any other endpoints. Curiously, arterial hypertension was associated with significantly longer TTNC in univariate analysis and a trend toward longer TTNC and OS in multivariate analysis. This effect was noteworthy, and it warrants further investigation as there might be a hitherto unknown synergism with antihypertensive co‐medications and PLD.

Due to its retrospective nature, this study has relevant limitations. The overall sample size was small. Data regarding dosing of PLD, performance status, geriatric assessment, and patient‐reported outcomes such as quality of life were not available for systematic analysis. Also data regarding safety and tolerability were not retrospectively available; however, these could have been relevant factors contributing to short TTNC, especially considering an elderly population. Given our focus on vulnerable patients, it would have been desirable to analyze these variables. Finally, our study population was heterogeneous regarding treatment line, thus impeding comparability to trial data to some extent.

## 5 CONCLUSIONS

By investigating all patients treated with single‐agent PLD at our institution, we could show that real‐world results of PLD in patients with MBC cannot live up to previously published data from prospective clinical trials and observational studies. This is likely due to a sampling bias, and it must be assumed that the general morbidity is higher in real‐world patient populations than in the selected populations of clinical trials. Observed outcomes in older patients and patients with comorbidities were not relevantly inferior to the overall population. PLD therefore remains a valid treatment option in these more vulnerable patient groups. However, expectations should be managed.

## AUTHOR CONTRIBUTIONS

**Till Wallrabenstein:** Conceptualization (lead); data curation (lead); formal analysis (lead); investigation (lead); methodology (lead); project administration (equal); resources (equal); software (lead); supervision (equal); validation (equal); visualization (lead); writing – original draft (lead); writing – review and editing (lead). **Eveline Daetwyler:** Data curation (supporting); formal analysis (supporting); investigation (supporting); resources (supporting); validation (supporting); writing – original draft (supporting). **Anton Oseledchyk:** Data curation (supporting); formal analysis (supporting); validation (supporting); visualization (supporting); writing – review and editing (supporting). **Christoph Rochlitz:** Conceptualization (supporting); investigation (supporting); methodology (supporting); validation (supporting); writing – review and editing (supporting). **Marcus Vetter:** Conceptualization (lead); data curation (supporting); formal analysis (equal); investigation (equal); methodology (equal); project administration (lead); resources (equal); supervision (lead); validation (equal); writing – original draft (equal); writing – review and editing (lead).

## FUNDING INFORMATION

None.

## CONFLICT OF INTEREST STATEMENT

The authors declare that they have no known competing financial interests or personal relationships that could have appeared to influence the work reported in this paper.

## ETHICS APPROVAL

This study was performed in line with the principles of the Declaration of Helsinki. Approval was granted by the responsible local ethics committee, Ethikkommission Nordwest‐und Zentralschweiz (EKNZ, Switzerland) on July 21, 2021 (project ID 2021–00709).

## CONSENT TO PARTICIPATE

For this type of study formal consent is not required. Nevertheless, informed consent was obtained from most participants included in the study. Due to the retrospective nature of this study, consent could not be obtained from all patients (e.g., deceased patients) and in these cases, the requirement for written consent was explicitly waived by the ethics committee.

## Supporting information


Table S1.
Table S2.
Table S3.
Table S4.

## Data Availability

The data that support the findings of this study are available on request from the corresponding author. The data are not publicly available due to privacy and ethical restrictions.
