# Supplementary material for: Pegylated liposomal doxorubicin (PLD) in daily practice—A single center experience of treatment with PLD in patients with comorbidities and older patients with metastatic breast cancer
Source: Cancer Med. 2023 May 6;12(12):13388–96. doi: 10.1002/cam4.6041 (PMC10315717; doi:10.1002/cam4.6041)
Supplement: Supplementary file 1 — Table S1. Table S2. Table S3. Table S4. [file CAM4-12-13388-s001.docx]

**SUPPLEMENTARY MATERIAL/APPENDIX**:

| Variabel | Median time to next chemotherapy or death (95% CI) | p | HR (95% CI) | p |
| --- | --- | --- | --- | --- |
| All | 4.6 (3.6-5.6) |  |  |  |
| Age >70 at treatment initiation (n=34) | 5.0 (0.0-10.4) | 0.36 | 0.88 (0.54-1.43) | 0.60 |
| Heart disease (n=10) | 7.0 (1.8-12.2) | 0.43 | 1.18 (0.53-2.63) | 0.69 |
| Hypertension (n=31) | 8.3 (6.3-10.3) | **0.04** | 0.62 (0.37-1.05) | 0.07 |
| ER positive (n=90) | 4.6 (1.8-7.4) | 0.5 | 0.72 (0.23-2.25) | 0.57 |
| PR positive (n=78) | 4.8 (1.8-7.8) | 0.54 | 0.88 (0.47-1.64) | 0.68 |
| Her-2 positive (n=11) | 4.6 (0.6-.8.5) | 0.46 | 1.36 (0.48-3.80) | 0.56 |
| TNBC (n=14) | 4.0 (1.9-6.0) | 0.77 | 0.73 (0.19-2.84) | 0.65 |
| Grade 3 (n=46) | 5.0 (0.9-9.1) | 0.58 | 1.02 (0.62-1.68) | 0.94 |
| Metachronous MBC (n=76) | 4.5 (3.5-5.5) | 0.68 | 1.24 (0.72-2.14) | 0.44 |
| Previous neo-/adjuvant anthracycline (n=34) | 4.7 (3.9-5.5) | 0.97 | 0.66 (0.33-1.31) | 0.24 |
| Previous neo-/adjuvant taxane (n=27) | 4.0 (1.9-6.1) | 0.1 | 1.68 (0.85-3.35) | 0.14 |
| Previous endocrine treatment for MBC (n=69) | 4.5 (2.9-6.0) | 0.61 | 1.16 (0.65-2.07) | 0.62 |
| Any previous chemotherapy for MBC (n=60) | 4.6 (3.2-6.0) | 0.83 | 1.23 (0.66-2.23) | 0.52 |
| Previous taxane for MBC (n=41) | 4.7 (3.2-6.2) | 0.57 | 0.73 (0.39-1.38) | 0.33 |

**Supplementary Table 1**: Univariate (logrank) and multivariate analysis (Cox regression) investigating patient-related, disease-related and treatment-related factors for predictive impact on the primary endpoint (time to initiation of next subsequent chemotherapy or death) in 112 patients with metastatic breast cancer treated with single-agent pegylated liposomal doxorubicin (PLD).

*CI=confidence interval, ER=estrogen receptor, Her2=human epidermal growth factor receptor 2, HR=hazard ratio, MBC=metastatic breast cancer, n=number, p=p-value, PR=progesterone receptor, TNBC=triple negative breast cancer, TTN=time to initiation of next subsequent treatment line.*

| Variabel | Median OS (95% CI) | p | HR (95% CI) | p |
| --- | --- | --- | --- | --- |
| All | 11.9 (1.0-13.9) |  |  |  |
| Age > 70 years (n=34) | 11.2 (7.9-14.5) | 0.23 | 1.83 (1.07-3.11) | **0.026** |
| Heart disease (n=10) | 3.9 (0.7-16.0) | 0.48 | 1.51 (0.68-3.39) | 0.31 |
| Hypertension (n=31) | 16.0 (12.1-19.9) | 0.14 | 0.63 (0.36-1.10) | 0.10 |
| ER positive (n=90) | 12.7 (10.6-14.7) | 0.1 | 1.01 (0.26-3.86) | 0.99 |
| PR positive (n=78) | 12.9 (10.8-14.9) | **0.05** | 0.57 (0.29-1.12) | 0.10 |
| Her-2 positive (n=11) | 13.0 (9.5-16.6) | 0.88 | 0.72 (0.22-2.35) | 0.59 |
| TNBC (n=14) | 8.6 (1.5-15.7) | 0.08 | 0.82 (0.17-4.02) | 0.80 |
| Grade 3 (n=46) | 12.9 (10.6-15.2) | 0.92 | 0.90 (0.55-1.47) | 0.67 |
| Metachronous MBC (n=76) | 11.8 (8.2-15.4) | 0.44 | 0.72 (0.40-1.29) | 0.27 |
| Previous neo-/adjuvant anthracycline (n=34) | 11.8 (9.5-14.1) | 0.3 | 1.09 (0.53-2.24) | 0.81 |
| Previous neo-/adjuvant taxane (n=27) | 10.1 (5.7-14.6) | 0.11 | 1.74 (0.86-3.53) | 0.13 |
| Previous endocrine treatment for MBC (n=69) | 13.4 (10.9-15.9) | 0.78 | 0.91 (0.50-1.66) | 0.76 |
| Any previous chemotherapy for MBC (n=60) | 11.7 (9.9-13.6) | 0.72 | 0.80 (0.41-1.56) | 0.52 |
| Previous taxane for MBC (n=41) | 10.6 (7.8-13.3) | 0.07 | 1.64 (0.82-3.29) | 0.16 |

**Supplementary Table 2**: Univariate (logrank) and multivariate analysis (Cox regression) investigating patient-related, disease-related and treatment-related factors for predictive impact on the secondary endpoint overall survival in 112 patients with metastatic breast cancer treated with single-agent pegylated liposomal doxorubicin (PLD).

*CI=confidence interval, ER=estrogen receptor, Her2=human epidermal growth factor receptor 2, HR=hazard ratio, MBC=metastatic breast cancer, n=number, OS=overall survival, PR=progesterone receptor, TNBC=triple negative breast cancer.*

| Variabel | Median PFS (95% CI) | p | HR (95% CI) | p |
| --- | --- | --- | --- | --- |
| All | 4.4 (3.3-5.5) |  |  |  |
| Age > 70 years (n=34) | 4.4 (3.0-5.8) | 0.53 | 1.39 (0.86-2.25) | 0.18 |
| Heart disease (n=10) | 5.2 (1.5-8.9) | 0.2 | 0.63 (0.28-1.42) | 0.26 |
| Hypertension (n=31) | 6.8 (4.5-9.1) | 0.4 | 0.82 (0.49-1.37) | 0.45 |
| ER positive (n=90) | 4.2 (2.6-5.8) | 0.98 | 1.33 (0.39-4.48) | 0.65 |
| PR positive (n=78) | 5.0 (3.2-6.8) | 0.38 | 0.71 (0.39-1.29) | 0.26 |
| Her-2 positive (n=11) | 6.8 (5.1-8.5) | 0.4 | 0.65 (0.21-1.99) | 0.45 |
| TNBC (n=14) | 2.8 (0.2-5.4) | 0.62 | 0.99 (0.25-3.93) | 0.99 |
| Grade 3 (n=46) | 4.0 (1.5-6.6) | 0.79 | 1.17 (0.73-1.87) | 0.52 |
| Metachronous MBC (n=76) | 3.8 (2.3-5.3) | 0.38 | 1.10 (0.65-1.86) | 0.72 |
| Previous neo-/adjuvant anthracycline (n=34) | 4.0 (2.6-5.4) | 0.85 | 0.70 (0.35-1.40) | 0.31 |
| Previous neo-/adjuvant taxane (n=27) | 3.6 (1.7-5.5) | 0.13 | 1.68 (0.85-3.32) | 0.14 |
| Previous endocrine treatment for MBC (n=69) | 4.2 (1.9-6.5) | 0.93 | 0.97 (0.56-1.68) | 0.91 |
| Any previous chemotherapy for MBC (n=60) | 4.8 (2.4-7.2) | 0.87 | 0.83 (0.46-1.5) | 0.54 |
| Previous taxane for MBC (n=41) | 4.7 (2.7-6.7) | 0.55 | 1.49 (0.80-2.80) | 0.21 |

**Supplementary Table 3**: Univariate (logrank) and multivariate analysis (Cox regression) investigating patient-related, disease-related and treatment-related factors for predictive impact on the secondary endpoint progression free survival in 112 patients with metastatic breast cancer treated with single-agent pegylated liposomal doxorubicin (PLD).

*CI=confidence interval, ER=estrogen receptor, Her2=human epidermal growth factor receptor 2, HR=hazard ratio, MBC=metastatic breast cancer, n=number, OS=overall survival, PR=progesterone receptor, TNBC=triple negative breast cancer.*

| Variabel | Median time to next treatment line (including endocrine) or death (95% CI) | p | HR (95% CI) | p |
| --- | --- | --- | --- | --- |
| All | 4.6 (3.7-5.5) |  |  |  |
| Age > 70 years (n=34) | 4.9 (1.1-8.7) | 0.21 | 0.79 (0.49-1.29) | 0.35 |
| Heart disease (n=10) | 7.0 (1.8-12.2) | 0.58 | 1.21 (0.56-2.62) | 0.63 |
| Hypertension (n=31) | 7.1 (2.7-11.5) | 0.054 | 0.64 (0.38-1.06) | 0.08 |
| ER positive (n=90) | 4.5 (3.3-5.7) | 0.75 | 0.61 (0.20-1.84) | 0.38 |
| PR positive (n=78) | 4.7 (3.3-6.1) | 0.99 | 1.09 (0.58-2.06) | 0.79 |
| Her-2 positive (n=11) | 4.6 (0.6-8.5) | 0.63 | 1.10 (0.41-2.98) | 0.85 |
| TNBC (n=14) | 4.0 (1.9-6.0) | 0.55 | 0.52 (0.14-1.96) | 0.34 |
| Grade 3 (n=46) | 4.6 (2.6-6.6) | 0.83 | 1.27 (0.77-2.08) | 0.34 |
| Metachronous MBC (n=76) | 4.3 (3.1-5.5) | 0.66 | 1.17 (0.68-2.02) | 0.57 |
| Previous neo-/adjuvant anthracycline (n=34) | 4.6 (3.8-5.4) | 0.9 | 0.67 (0.35-1.72) | 0.24 |
| Previous neo-/adjuvant taxane (n=27) | 4.0 (1.9-6.1) | 0.21 | 1.49 (0.78-2.00) | 0.23 |
| Previous endocrine treatment for MBC (n=69) | 4.5 (3.4-5.5) | 0.39 | 0.97 (0.54-1.60) | 0.90 |
| Any previous chemotherapy for MBC (n=60) | 4.6 (3.6-5.5) | 0.96 | 1.06 (0.56-1.5) | 0.86 |
| Previous taxane for MBC (n=41) | 4.6 (3.7-5.6) | 0.56 | 0.84 (0.44-2.80) | 0.59 |

**Supplementary Table 4**: Univariate (logrank) and multivariate analysis (Cox regression) investigating patient-related, disease-related and treatment-related factors for predictive impact on the secondary time to initiation of next subsequent therapy or death in 112 patients with metastatic breast cancer treated with single-agent pegylated liposomal doxorubicin (PLD).

*CI=confidence interval, ER=estrogen receptor, Her2=human epidermal growth factor receptor 2, HR=hazard ratio, MBC=metastatic breast cancer, n=number, OS=overall survival, PR=progesterone receptor, TNBC=triple negative breast cancer.*
